# Percutaneous osteoplasty for the management of a pubic bone metastasis

**DOI:** 10.1007/s00132-019-03708-7

**Published:** 2019-03-06

**Authors:** Guan Shi, Hai Tang

**Affiliations:** Department of Orthopedics, Beijing Friendship Hospital, Capital Medical University No. 95, Yong An Rd, Xi Cheng District, 100050 Beijing, China

**Keywords:** Pelvis, Carcinoma, transitional cell, Osteolysis, Polymethyl methacrylate, Case report, Becken, Transitionalzellkarzinom, Osteolyse, Polymethylmethacrylat, Fallbericht

## Abstract

Percutaneous osteoplasty (POP) has been proven to relieve pain due to osteolytic metastases by injecting bone cement to stabilize the pathological fracture. Nevertheless, there have been few reports about POP of metastases in the pubis. This article presents a case involving the use of POP to manage a metastasis in the pubis. After POP the patient experienced significant pain relief and improvement in the quality of life.

## Zusammenfassung

Die perkutane Osteoplastik (POP) lindert nachweislich Schmerzen, die durch osteolytische Metastasen hervorgerufen werden, indem zur Stabilisierung pathologischer Frakturen Knochenzement injiziert wird. Gleichwohl gab es einige Berichte über POP bei Metastasen im Schambein. In diesem Beitrag wird ein Fall vorgestellt, in dem die POP zur Behandlung einer Metastase im Schambein verwendet wurde. Nach der POP hatte der Patient deutlich weniger Schmerzen, und seine Lebensqualität verbesserte sich.

## Introduction

The pelvis is a vulnerable site for osteolytic malignant tumors [1, 2]. The treatment of skeletal metastases is mainly dependent on radiotherapy, chemotherapy or hormone therapy. In most cases, the pain caused by osteolytic metastases can be relieved by conservative treatment, such as radiotherapy and oral opioids [3, 4]; however, unlike other skeletal metastases, pubic metastases usually cause such severe pain due to invasion of the peripheral nerves that painkillers cannot achieve a satisfactory effect.

Percutaneous osteoplasty (POP) is a minimally invasive procedure widely used in skeletal metastases by injecting bone cement to stabilize the micropathological fracture. It has been proven to be effective to relieve pain in metastases of vertebrae, femur, humerus and the sacrum [5–9]. Nevertheless, there have only been few reports about the use of POP in pubic metastases [10–12]. Therefore, this article is mainly to report the case of one patient with pubic metastases treated with POP in this hospital.

## Case report

A 67-year-old female patient underwent a computed tomography (CT) scan because of constant pain in the left hip. The result revealed a left-sided transitional cell carcinoma of the renal pelvis. Multiple metastatic bone tumors were diagnosed by bone scan with a high metabolism of 18F-fluorodeoxyglucose (18-FDG) in the fifth lumber vertebra (L5), left ischium and pubis. In addition, the patient suffered from hypertension, coronary atherosclerosis with a cardiac stent and a pacemaker. The patient received regular radiotherapy and chemotherapy to treat metastases and took opioids to relieve the pain; however, the constant pain could not be relieved after 1 month of oral painkiller administration. Repeated pain severely affected the patient’s sleeping and quality of life. Given the patient’s short life expectancy and the potential of POP to rapidly relieve pain and improve the function of limb activity, an individual treatment plan was developed. First, percutaneous vertebroplasty (PVP) was performed to treat the L5 tumor. Then, POP was performed to treat pubic bone metastases. After surgery, the patient received regular treatment with radiotherapy, chemotherapy and zoledronic acid. Informed consent about the possible benefits and risks of treatment was signed by the patient and her family.

### Interventional technique

First, the patient underwent PVP of the L5 tumor when lying in a prone position. Then, the patient was changed to the lithotomy position to undergo POP on the pubic bone metastases. The puncture needle point was located and marked under the C‑arm X‑ray. Local anesthesia was induced after sterilizing the skin of operation area and paving the sterile towels. Third, a needle (Kyphon®, Medtronic, CA, USA) was used to puncture the superior pubic ramus. It was adjusted by the sagittal and axial images of C‑arm X‑ray until it reached the tumor. The needle was removed and polymethyl methacrylate (PMMA, Osteopal V, Heraeus Medical, Wehrheim, Germany) was slowly injected into the tumor under the projection of C‑arm X‑ray. The injection was stopped when the metastatic tumor was completely filled with PMMA. The same operation process was applied to inject 3 ml of PMMA into the lower branch of the pubic metastases.

**Fig. 1:**
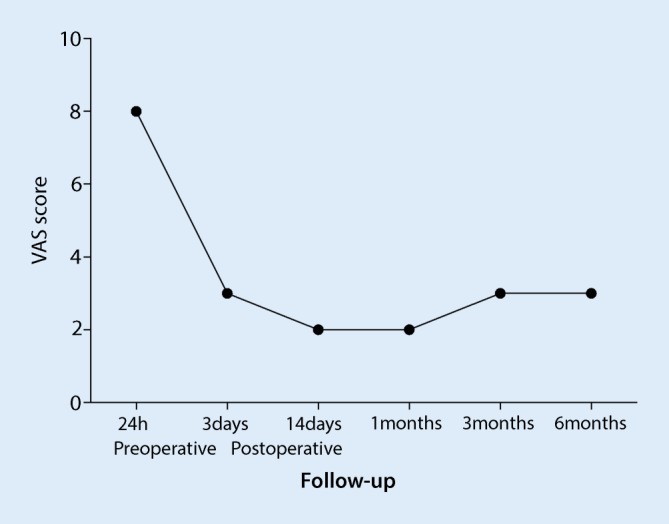
Graph shows the changes in the visual analogue scale score of the patient from preoperative to 6 months postoperative

**Fig. 2:**
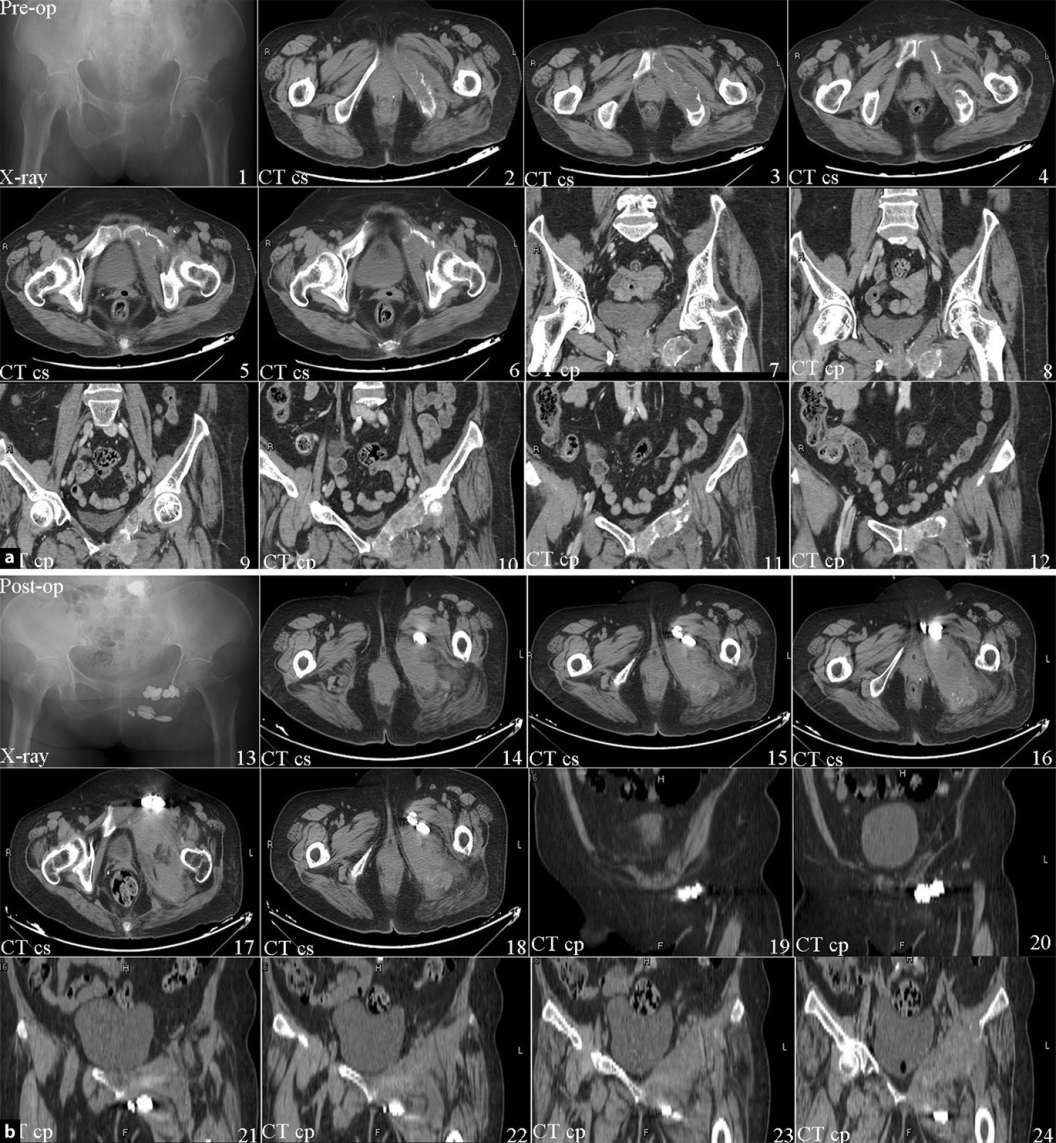
**a** *Pre-op* preoperative, **b** *post-op* postoperative. The preop CT shows the bone surface was sufficiently intact. The postop CT shows the bone cement filled the metastatic lesion and extruded into the soft tissues. *CT* *cs* computed tomography cross-section, *CT* *cp* computed tomography coronal plane

During surgery, the patient’s blood pressure, electrocardiogram pattern and pulse oximetry waveforms were all stable. Complications such as limb nerve injury, bleeding, embolism and infection did not occur during and after surgery. During the following 3 days, the patient was able to fall asleep without feeling pain. The VAS score decreased from preoperative 8 to postoperative 3. After 6 months the VAS was stable at 2. Details of the VAS are shown in Fig. 1. Preoperative and postoperative images are showed in Fig. 2.

## Discussion

The pelvic ring is composed of the ilium, sacrum, pubis and ischium. It is surrounded by bundles of blood vessels and nerves of the lower extremities. Thus, it is very difficult to completely resect tumors in the pelvic ring because they often invade the circumambient vital organs. Even if the tumor is resected, the recurrence rate is as high as 40–70% and the 5‑year tumor-free survival rate is less than 30%. Therefore, malignant tumors including metastases in the ilium, sacrum, pubis and ischium have been the greatest challenge for doctors for many years [13, 14]. For a long time, the main surgical method for pelvic malignancies was hemipelvectomy. Although this traditional surgical method may partially reduce the local recurrence of the tumor, the patient lost the hemilateral pelvis and the ipsilateral lower limb. In recent years, due to the development of radiotherapy, chemotherapy and surgical techniques, limb salvage surgery is widely applied to pelvic malignancies. Especially for acetabulum metastases with severe pain and difficulty walking, complete metastases resection and limb function reconstruction may be the best way to restore hip mobility and stability. Before surgery it is necessary to evaluate the patient’s general condition, the nature of the tumor, the possible survival time, the number of metastases and the extent of the lesion. If the malignant metastases present a good clinical prognosis, such as kidney cancer, breast cancer and thyroid cancer patients with long-term survival, surgical treatment should be actively taken to improve the quality of life of patients [15–17]. In the present study, except for pubic bone metastases, the patient suffered from lumbar vertebrae metastases and coronary atherosclerosis with a cardiac stent and a pacemaker, so extensive pubic bone resection was not one of the best treatment options.

While POP is a minimally invasive treatment, it may quickly stabilize the pubic pathological fracture by injection of bone cement. The corrected stability may prevent the osteolytic bones from continuing to deform by relieving the pressure on the painful periosteum [18]. In addition, there are several possible mechanisms of pain relief in patients with metastases treated with POP. First, PMMA may reduce the stimulation to nerve endings by stabilizing the pathological fracture caused by osteolytic metastases. Second, the toxic effect of PMMA monomer may weaken the ability of nerve endings to transmit the sense of pain. Third, the exothermic reaction of PMMA polymerization may destroy nerve endings and play a role as an anti-tumor agent [19–21].

During the follow-up period, the patient’s pain and quality of life clearly improved. Especially after post-procedure day 3, the pain and lower limb function greatly improved to a stabilized situation. Therefore, POP is an effective treatment and can be used as an alternative method to treat pubic bone metastases. The procedure of POP is similar to PVP, which involves a needle being inserted into metastatic tumors and injecting PMMA. Before the operation, it is necessary to carefully analyze the patient’s images to identify the extent of metastases. During the operation, C‑arm X‑ray should be used to accurately locate the puncture site and monitor the process of injecting PMMA to avoid bone cement leakage. After the operation, the patient received regular treatment of chemotherapy and zoledronic acid. The follow-up showed that POP combined with chemotherapy presented a promising effect on pubic bone metastases.

## Limitation

Although POP achieved a satisfactory result in this case, it is necessary to conduct a large study of many patients to further validate the efficacy and to clarify the surgical indications and related complications.

## Abbreviations

CT: Computed tomography
18FDG: 18-Fluorodeoxyglucose
L5: Fifth lumbar vertebra
PMMA: Polymethyl methacrylate
POP: Percutaneous osteoplasty
PVP: Percutaneous vertebroplasty
VAS: Visual analogue scale
